# Impact of the Urology Assessment Unit on the Emergency Urology Patient Pathway

**DOI:** 10.7759/cureus.94293

**Published:** 2025-10-10

**Authors:** Hasan A Al-Ibraheem, Nadine McCauley, Mudassir Wani, Jim Wilson

**Affiliations:** 1 Urology, Aneurin Bevan University Health Board, Newport, GBR

**Keywords:** acute urology, emergency urology, urology assessment unit, urology patients, urology referral

## Abstract

Objective

A Urology Assessment Unit (UAU) was developed to allow direct access for emergency urology patients. This study compared the new UAU patient pathway with the previous Surgical Assessment Unit (SAU), looking at the source and type of referrals, the time to assessment and decision-making for patients' discharge or admission to hospital from the unit.

Subjects/patients (or materials) and methods

Retrospective data collection and analysis using admission diaries and online patient portals were conducted for four weeks for patients seen in the SAU (October-November 2019) and a four-week period after the establishment of the UAU (September 2023) at the Aneurin Bevan University Health Board in Caerleon, Newport, Wales.

Results

A total of 207 patients attended the UAU over four weeks, compared to 111 in the SAU. The UAU had more patient self-referrals, 44 patients (21%), while in the SAU, there were five patients (4.5%). District nurses referred 15 patients (7%) to the UAU and none to the SAU. In the UAU, 104/186 (55.9%) patients were seen within 30minutes.

Conclusion

The UAU was associated with a higher number of urology patients seen and with shorter documented waiting times, with prompt management decisions made with the senior urology input. Advanced nurse practitioners (ANPs) are allowed to support in managing common urological presentations. The sources of patient referrals changed with increased district nurse referrals and patient self-referrals to the UAU.

## Introduction

The Urology Assessment Unit (UAU) was set up in 2020 for emergency urology patients to have direct access to the emergency urology team at a district general hospital. Originally, these patients had been attending the Surgical Assessment Unit (SAU) alongside all other surgical specialties. This development was partly logistical, with an organisational change leading to urology being the only surgical specialty based at one hospital site.

The design of the UAU reflected a change in the staffing of the emergency urology team, with Advanced Nurse Practitioners (ANPs) now available as part of the emergency urology take team. The ANPs underwent focused training for urological presentations and undertook a Master's in Clinical Practice as part of their role. Urology surgical house officers (SHOs) or resident doctors are based at the UAU 24 hours a day, seven days a week. They are supervised by the urology registrar or senior resident doctor on call, as well as a dedicated consultant urologist free of other clinical commitments, completing the on-call team. The UAU is staffed with at least one trained nurse from the attached urology inpatient ward. The unit has a waiting area, four trolley areas for assessment, and is stocked with specialised equipment to manage common acute urological presentations, such as specialised urethral catheters, portable cystoscopes, and bladder scanners.

With an estimated 132,000 emergency admissions a year to hospitals in the United Kingdom under the care of a consultant urologist [1], the impact of a dedicated urology team and place to assess, treat, and manage the emergency urology patient pathway is explored in this study.

## Materials and methods

This study was undertaken in the Aneurin Bevan University Health Board (Caerleon, Newport, Wales), through retrospective data collection for SAU patients who attended the unit during two time periods: from October 1, 2019, to October 14, 2019, and from November 28, 2019, to December 11, 2019. These discrete windows were selected because complete SAU diary records were available for these intervals. Patients were included based on records kept in SAU diaries for all admissions to the unit during those time periods, and the urology presentations were included in the data collection. Patients who attended the UAU between September 1, 2023, and September 28, 2023, were included in the UAU period for comparison. There is a four-year gap because there was a transition period from the SAU to the UAU, and to avoid the pandemic's effect on our analysis. 

All the urology patients in the mentioned time frames in both units were included, and no exclusion criteria were applied. Once these patients were identified, data collection was performed using online patient information portals and by reviewing scanned admission documents. Data were collected using Microsoft Excel software (Microsoft Corp., USA), anonymised, and kept on a secure computer. Data were evaluated, and results were interpreted accordingly. No further statistical analysis was carried out due to the data being epidemiological and the study being observational. 

The primary aim was to compare the time from arrival to first documented clinical contact by a urology clinician (ANP, SHO, registrar, or consultant) and the total time from arrival to documented discharge or hospital admission between SAU and UAU cohorts. The secondary aim of the study was to investigate patient demographics and the source of referrals.

## Results

During the two study periods, 111 patients were seen in the SAU and 207 patients in the UAU. The age range of patients in both units was between 16 and 99 years, with a median age of 67 and a mean age of 63.57. The male-to-female ratio was 81% to 19% with 258 males and 60 females.

The most common source of referrals in both units was general practitioners (GPs), who made up 58 (52%) of the referrals to the SAU and 57 (27.5%) to the UAU. The second most common referral source to the SAU was from the Accident and Emergency Department (A&E), with 28 (25%) patients, while in the UAU, it made up 43 referrals (20.7%). Patient self-referrals to the UAU were 44 (21%), while in the SAU, these made up five (4.5%) referrals. Reflecting the change of urology patient pathways into the hospital, district nurse referrals numbered 15 to the UAU (7%), with none received directly to the SAU.

**Figure 1:**
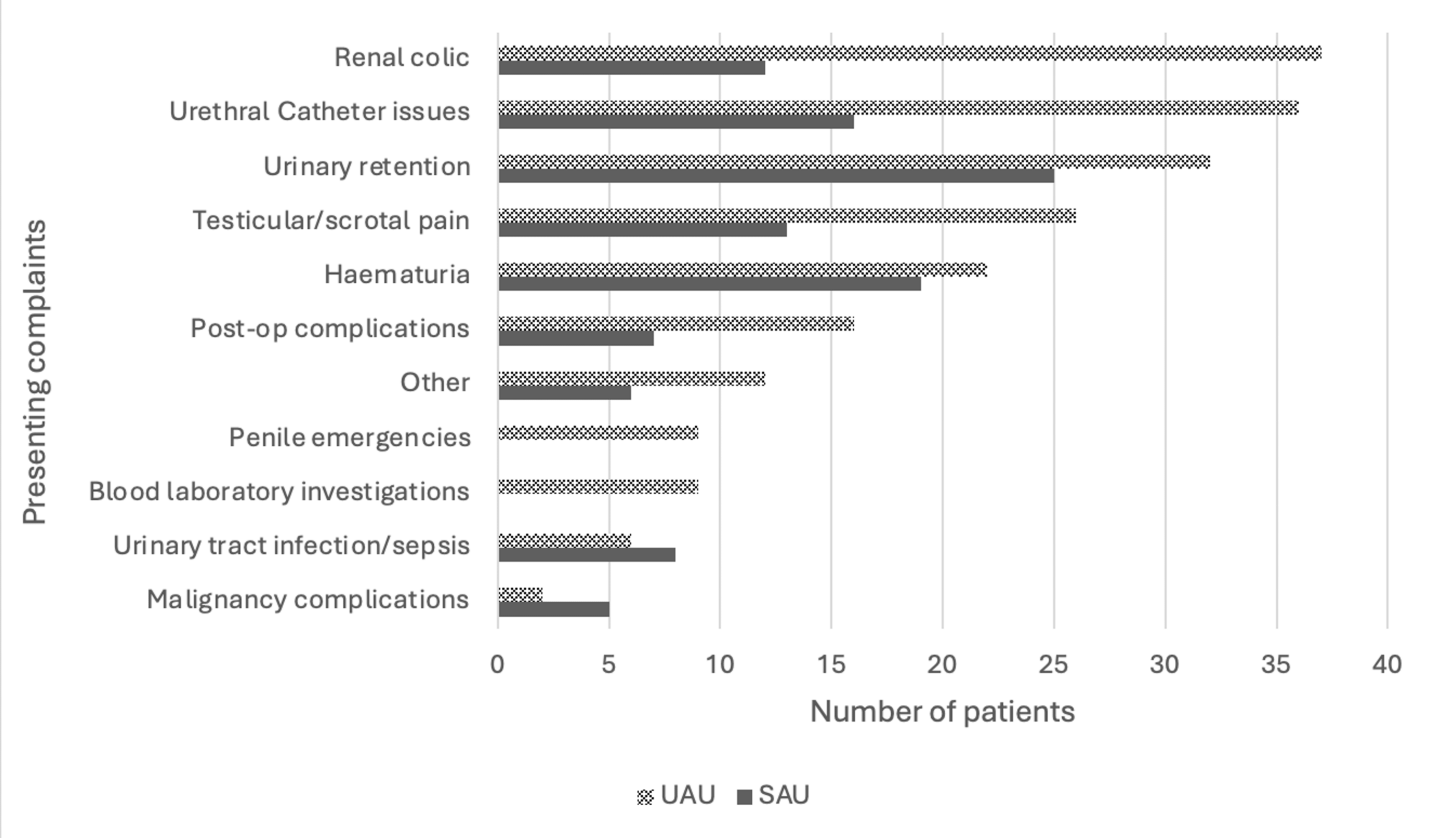
Presenting complaints of the patients attending SAU and UAU. Surgical Assessment Unit (SAU): n = 111; Urology Assessment Unit (UAU): n = 207

The most common urological presentation to the SAU was urinary retention in 25 (22.5%) patients, which made up 32 (15.4%) patients assessed in the UAU. Among UAU presentations, renal colic was the most common, with 37 (17.8%) patients. The distribution of other presenting complaints to the SAU and UAU is demonstrated in Figure 1.

**Figure 2:**
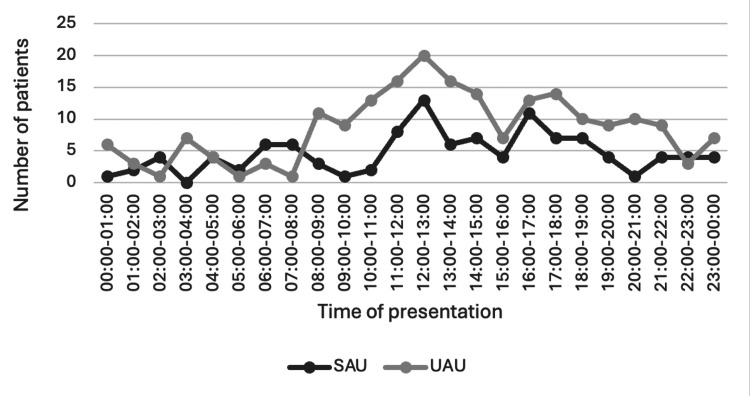
Time of presentation of patients to the SAU and UAU. Surgical Assessment Unit (SAU): n = 111; Urology Assessment Unit (UAU): n = 207

Both units were accessible to patients 24 hours per day, seven days a week. The time of patient presentation to each unit is demonstrated in Figure 2. The peak hour of arrival for patients to both the SAU and UAU was between 12:00 and 13:00 hours, with a minority of patients arriving out of hours between 20:00 and 08:00, 38 (34%) patients in the SAU and 55 (26.5%) in the UAU. Same‑day discharge occurred in 158/207 (76.3%) UAU patients and 76/111 (68.5%) SAU patients. The remainder were admitted as urology inpatients. “Same‑day discharge” was defined as documented discharge from the assessment unit on the day of presentation with either outpatient follow-up or discharge back to the general practitioner care.

Part of the design of UAU was the inclusion of the role of an ANP trained to review common urological presentations. Eight (3.9%) patients were reviewed and managed solely by an ANP during the study period. Eighty-seven (42%) patients were only assessed and discharged from the UAU by senior house officers (SHOs). Seventy-three (35.2%) patients needed a urology specialty registrar review, and 37 (17.8%) patients needed a consultant review.

**Figure 3:**
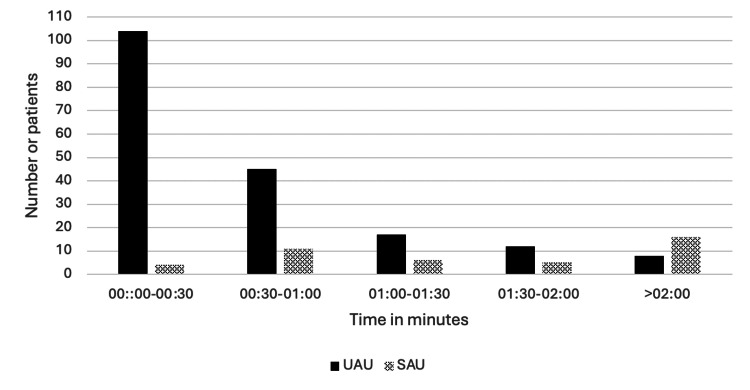
Distribution of patients' waiting times to be seen by a clinician in the UAU and SAU. Surgical Assessment Unit (SAU): n = 42 (data available); Urology Assessment Unit (UAU): n = 186

The mean time from arrival to first documented clinical contact by a urology clinician (ANP, SHO, registrar, or consultant) was 41 minutes (date available for 186/207 patients) in UAU and two hours and 18 minutes (data available for 42/111 patients) in the SAU. Moreover, 104/186 (55.9%) of the UAU patients were seen within 30 minutes. In the SAU, 4/42 (9.5%) of the urology patients were seen within 30 minutes by a urology clinician. In the SAU, 16/42 (38.1%) of patients with available timing data waited more than two hours to be seen, compared with 4/186 (2.2%) in UAU (Figure 3).

The mean total time spent by patients before discharge or admission, where information was available, was three hours 15 minutes (n = 204) in the UAU and six hours 59 minutes (n = 110) in the SAU. Moreover, 86/204 (42%) of the patients stayed in the UAU for less than two hours. National guidelines for A&E waiting times are less than four hours [2]. During the study period, it was noted that 65/110 (59%) of the patients waited in the SAU for over four hours, whereas this was the case for 62/207 (29.9%) of the patients in the UAU.

## Discussion

Urology patients in the United Kingdom presenting to the hospital as an emergency are commonly assessed in Accident and Emergency or a unit dedicated to surgical emergencies, such as the SAU [3]. The SAU was initially designed to assess acute surgical patients and urology patients in most of the UK hospitals, relieving some of the workload of A&E departments, improving patient care, and decreasing patient waiting times [3-5]. There have, however, been limited descriptions in published literature of a specialised urological assessment unit. One published article suggested that 93% of patients preferred to attend the UAU directly rather than accessing A&E or GP as a primary route [6].

This study demonstrated an increase of almost double the number of urology patients being seen by the urology team over a four-week period following the introduction of the UAU. In 2019, 111 patients were referred to Urology, whereas in 2023, this had risen to 207 patients during the same length of time. This may reflect an increasing workload for urology teams and suggests the need for greater future workforce capacity to manage an increasing volume of patients. One area of change was the increased referrals to the UAU from district nurses; a local agreement had been made to allow district nurses to refer urethral catheter issues directly to the UAU rather than via the GP or A&E. This is reflected in the data, where no district nurse referrals were received direct to the SAU, while 7% of UAU referrals were from the district nurse community teams.

Another change to the source of referral was the increased incidence of patient self-referral to the UAU. In particular, 24% of UAU admissions came from patients referring themselves for direct urological specialist review in comparison with 5% seen in the SAU previously. Contact information for the UAU is given to elective urology patients in the postoperative setting as part of the local safety netting process. This allows for increased patient access to self-refer with issues related to their recent admission to the hospital. A previous study of patient preferences for accessing a service such as the UAU commented that urology patients appreciated seeing a specialist directly, being given the opportunity to avoid attending their GP or A&E departments as the primary route of care [6].

In the UK, there has been an increased emphasis on treating patients appropriately with each interaction through the Getting it Right First Time (GIRFT) project [1]. This study reflects the GIRFT report, which lists the most common urological emergencies as urinary retention and renal colic, with these making up a third of the presentations to UAU. This report also recommends the role of specialised nurses in managing urological conditions to relieve the load on doctors, especially consultant urologists, to deliver timely emergency care [1]. Furthermore, 17% of referrals to the UAU were related to urethral catheter issues; this is a potential area for nurses with specialist urological experience and advanced catheter skills to be able to manage these referrals without a doctor’s involvement.

The time of arrival of patients in UAU was quite similar when compared with SAU, with both hitting a peak at midday. The least busy times were between 22:00 hours and 08:00 hours, likely reflecting the working hours of the community teams (GPs and district nurses). Despite the overall increase in patients attending the UAU, most patients waited a shorter amount of time, with 56% being seen within 30 minutes in the UAU, compared with 9.5% in the SAU. This is potentially reflective of the urology workforce changes accompanying the implementation of the UAU, with increased dedicated staffing of ANPs and on-call doctors to the unit. Furthermore, the presence of senior doctors and consultant urologists relieved of elective commitments may have contributed to swift decision making, with 42% of patients either being discharged or admitted within two hours of arrival, whereas the mean total time a patient stayed in SAU at almost seven hours.

This study has several limitations. First, it was retrospective in design and therefore dependent on the accuracy and completeness of admission paperwork and electronic records. Second, the analysis was limited to two short four-week study periods, which may not capture seasonal variation or longer-term patterns in emergency urology presentations. Third, as a single-centre study, the findings may not be directly generalisable to other hospitals with different referral systems, staffing models, or patient populations. In addition, clinical outcomes, including complications, readmissions, or patient satisfaction, were not evaluated. Data on waiting times in the SAU group were not available for all the patients, which may limit the strength of the direct comparison with UAU. Finally, there is a four-year interval between the SAU and UAU sampling periods, which may introduce temporal confounding.

## Conclusions

The introduction of a UAU with appropriate staffing is associated with an increased volume of urology patients to be seen in the unit, diverting this burden away from A&E and the SAU. Urology patients are seen quickly, and management decisions are made promptly with a senior urology input. The introduction of ANPs allows for support in managing common urological presentations. Patients are increasingly seeking specialist urological care through self-referral.
